# Molecular pathological epidemiology: the role of epidemiology in the omics-era

**DOI:** 10.1007/s10654-015-0093-7

**Published:** 2015-10-14

**Authors:** M. Arfan Ikram

**Affiliations:** Departments of Epidemiology, Radiology, Neurology, Erasmus Medical Center, PO Box 2040, 3000 CA Rotterdam, The Netherlands

The ‘omics’-era is well and truly upon us. Driven by technological innovations, fields such as genetics, epigenetics, proteomics and imaging are advancing at tremendous pace and in addition to unraveling pathophysiological mechanisms of health and disease, also reveal the increasingly complex biology that forms the basis of living systems. In this issue of the *European Journal of Epidemiology*, Nishihara and colleagues describe ‘molecular pathological epidemiology’, a relatively novel sub-field within epidemiology that examines the association between a risk factor and disease by taking into account the inherent pathological (and often molecular) heterogeneity of a disease [1]. Molecular pathological epidemiology had its original applications in oncology, but—driven by omics-research—is now quickly disseminating to other fields of biomedical research. Molecular pathological epidemiology holds great promise by bringing together epidemiology and molecular biology, two fields that traditionally have been considered at the opposite extremes of the biomedical research spectrum. It is expected that in coming years molecular pathological epidemiology will importantly shape the way, in which omics-research is conducted and interpreted.

In their paper, Nishihara et al. [1] use molecular pathological epidemiology to elucidate biomedical paradoxes that hitherto were ascribed to methodological phenomena, such as selection bias, collider bias, unmeasured confounding and reverse causality. They show that paradoxical associations that have been found between a risk factor for disease and prognosis after disease-onset might in fact be reflecting true underlying biology rather than being an artifact of study design. While the focus of their paper is on examples that elegantly illustrate the application of molecular pathological epidemiology to unravel methodological paradoxes, I believe this approach will have further-reaching implications in biomedical research, which are only briefly touched upon by Nishihara et al. and which I will expand on below.

First, I am convinced that molecular pathological epidemiology is a pivotal cornerstone for successful implementation of novel initiatives, such as ‘personalized medicine’ and its latest incarnation ‘precision medicine’. Precision medicine is probably an overhyped concept at the moment, but nonetheless does signal the direction in which clinical medicine should develop to reach the next level. The basic concept of precision medicine is that each individual has his or her own unique set of characteristics that together cause disease or determine prognosis. This unique set therefore requires a unique approach to clinical management, tailor-made for that individual patient [2]. The continued success of omics-research through novel discoveries will lead to rapid expansion of the known set of characteristics that determine disease and thereby increase the precision of precision medicine. Interestingly, this framework for disease causation was formalized in the epidemiological literature already as far back as the seventies [3]. As such, precision medicine is nothing new for epidemiology. In fact, the enthusiasm with which precision medicine has been heralded by various stake-holders and policy makers could and should be considered a vindication for the epidemiological way of thinking about disease. What is worrying though is that the role of the epidemiologist in precision medicine seems so far to be ill-defined. Yet, given the challenges that precision medicine faces for correct design and interpretation of its research (indeed, consider the paradoxical findings that can only be tackled by proper understanding of molecular pathological epidemiology and other epidemiological concepts), I would expect the epidemiologist to be at the fore-front of precision medicine. Therefore, now is the time for epidemiologists to assume a pro-active role in the process of developing and implementing precision medicine.

The second implication of molecular pathological epidemiology relates to a core activity in clinical medicine. Diagnosis of disease (and the efforts to achieve that) forms a major milestone in the clinical management of any patient and in fact much—if not most—effort in clinical practice is dedicated at establishing an accurate diagnosis. Diagnoses of diseases are usually based on a set of signs and symptoms that typically occur together in a patient and as such are considered a separate disease entity. Very often, diagnostic criteria also include the presumed underlying etiology, thereby forming the basis of etiologic subtyping. Examples include subtyping of ischemic stroke into large vessel, small vessel, or cardioembolic stroke and polyneurpathy into various subgroups, such as diabetic polyneuropathy. Such subclassifications have been developed for several decades and though very useful for contemporary clinical management, neglect the multifactorial nature of diseases [2]. Consequently, the underlying presumed etiology is neither sensitive nor specific enough to justify a single dedicated subcategory. For instance, most stroke patients will have a certain degree of small vessel pathology and it therefore remains arbitrary what burden of small vessel pathology warrants the designation of a stroke as small vessel stroke. Molecular pathological epidemiology can provide an etiologic framework for diseases, which does allow for etiologic subtyping. Taking examples from Nishihara et al. it can be justified to subclassify colorectal into two types based on presence or absence of the rs4939837 variant in the *SMAD7*-gene or subtyping renal cell carcinoma based on up- or downregulation of fatty acid synthase. After all, rs4939837 and up- or downregulation of fatty acide synthase may act as criteria sensitive and specific enough to distinguish the two subtypes. Omics-research will continue to further unravel many complex diseases and therefore expand the possibilities for etiological subtyping. Therefore, now is the time to re-write medical textbooks and incorporate molecular pathological epidemiology into mainstream biomedical literature.

Just like any other field, epidemiology too needs to keep up with the technology-push in research. Molecular pathological epidemiology might just be the trigger that helps epidemiology firmly establish its role in the omics-era.
